# Editorial: Cognitive benefits of technologies applied to learning in education

**DOI:** 10.3389/fpsyg.2025.1575482

**Published:** 2025-02-27

**Authors:** Beatriz Peña-Acuña, Rafael Crismán-Pérez, Yolanda Navarro-Abal, Pedro Román-Graván, Pedro Tadeu, Manuel León-Urrutia, Javier Ávila-López, Carmen M. Toscano-Fuentes, Carmen María Martín Del Pino

**Affiliations:** ^1^Faculty of Education, Psychology and Sports Sciences, University of Huelva, Huelva, Spain; ^2^Department of Spanish Language, Linguistics, and Literary Theory, Sevilla University, Seville, Spain; ^3^Department of Social, Developmental, and Educational Psychology, University of Huelva, Huelva, Spain; ^4^Faculty of Education, University of Seville, Seville, Spain; ^5^Center for Studies in Education and Innovation, Instituto Politécnico da Guarda, Guarda, Portugal; ^6^Department of Electronics and Computer Science, University of Southampton, Southampton, United Kingdom; ^7^Department of English and German Philology, University of Cordoba, Córdoba, Spain

**Keywords:** Cognitive, Technology, Benefits, Learning, Education

During the 2023/2024 academic year, a monographic Research Topic of the journal Frontiers has been published on the topic *Cognitive benefits of technologies applied to learning in education* (8,360 downloads and a total of 34 k downloads and views). At this point, two sections of the journal have received requests for publication: Frontiers in Education and Frontiers in Psychology. The team was composed of the following members:

1. Beatriz Peña Acuña (University of Huelva).
2. Carmen Martín del Pino (University of Huelva).
3. Carmen Toscano Fuentes (University of Huelva).
4. Francisco Javier Ávila López (University of Córdoba).
5. Pedro Tadeu (Polytechnic of Guarda Portugal).
6. Carmen del Pino (University of Huelva).
7. Yolanda Navarro Abal (University of Huelva).
8. Manuel León Urrutia (University of Southampton).
9. Pedro Roman Gravan (University of Seville).
10. Rafael Crismán Pérez (University of Seville).

A total of 84 applications for publication have been supervised and evaluated. This means that the total number of authors evaluated was 229. Of all the articles submitted, 17 applications were accepted, i.e., a percentage of approximately 20%. The titles are as follows:

Video games and metacognition in the classroom for the development of 21st century skills: a systematic review (Checa-Romero and Gimenez-Lozano).

The influence of mind mapping on computational thinking skills and self-efficacy in students' learning of graphical programming (Guo et al.).

Parents' rearing styles and adolescents' math achievement: the multiple mediating effect of self-control and math anxiety (Wang et al.).

The effect of augmented reality storybooks on the story comprehension and retelling of preschool children (Simşek).

Unlocking innovation: how enjoyment drives GenAI use in higher education (Cano and Nunez).

Toward a new model for the successful implementation of information and communication technologies in education (Lamalif et al.).

The integration of psychology and medicine: an empirical study of curriculum reform from the perspective of China (Ma et al.).

An isochronic substitution benefit study of the effects of screen time on the cognitive abilities of 3–6 children (Zhenya et al.).

Exploring the factors influencing high school students' deep learning of English in blended learning environments (Shi and Lan).

Prevalence of phubbing behavior in school and university students in Spain (Barbed-Castrejón et al.).

Ethnocultural empathy development of future language teachers through digital multiliteracy resources for low-literacy adult migrants (Fernández-Corbacho et al. a).

Immersive virtual reality for learning about ecosystems: effect of two signaling levels and feedback on action decisions (Porte et al.).

Shaping the future of creative education: the transformative power of VR in art and design learning (Serna-Mendiburu and Guerra-Tamez).

Educators as agents of breadth-biased learning: using social reconstructionism as rationale for embracing media multitasking and enhancing teaching practices in higher education (Kassie).

Gamification in the classroom: Kahoot! As a tool for university teaching innovation (Aibar-Almazán et al.).

Assessing the intention to accept inquiry-based teaching pedagogy among Chinese university students: an extension of technology acceptance model (Hu et al.).

The effect of self-directed online metacognitive listening practice on Chinese EFL learners' listening ability, metacognition, and self-efficacy (Pei et al.).

In addition to the two blocks (*Frontiers in Education* and *Frontiers in Psychology*), the following topics have been taken into account:

- *Cognition*.
- *Digital Education*.
- *Frontiers in Computer Science Mission statement*.
- *Digital Learning Innovations*.

Thus, after observing the accepted publications, we can see that the category of *Teaching and Learning Methods* presents more articles within the monograph. This category has covered issues such as students “attitudes toward hybrid learning in faculties of education, the comparison of audiovisual languages in the teaching of young and adult learners or the influence of the gamified approach on students” perception during the teaching and learning processes.

On the other hand, technology and its possibilities of use in education have raised two possibilities: *technology as a tool for transparency in education* and *the use of smartphones as a tool for teenagers*.

Finally, we will also highlight the importance of a cognitivist approach in second language teaching, as well as the topic of teacher training through digital resources. See Table 1 which lists the titles and category of each publication.

**Table 1:** List of articles.

**Title**	**Category**	**Type of research**
Video games and metacognition in the classroom for the development of 21st century skills: a systematic review (Checa-Romero and Gimenez-Lozano)	Teaching and Learning Methods	Systematic review
The influence of mind mapping on computational thinking skills and self-efficacy in students' learning of graphical programming (Guo et al.)	Educational technology and as a learning methodology	Quantitative (experimental)
Parents' rearing styles and adolescents' math achievement: the multiple mediating effect of self-control and math anxiety (Wang et al.)	Parenting and learning styles	Quantitative (cuasi experimental)
The effect of augmented reality storybooks on the story comprehension and retelling of preschool children (Simşek)	Teaching and Learning Methods	Quantitative (experimental)
Unlocking innovation: how enjoyment drives GenAI use in higher education (Cano and Nunez)	Educational technology and as a learning methodology	Quantitative (cuasi experimental)
Toward a new model for the successful implementation of information and communication technologies in education (Lamalif et al.)	Inclusion and educational equity	Systematic review
The integration of psychology and medicine: an empirical study of curriculum reform from the perspective of China (Ma et al.)	Teaching and Learning Methods	Quantitative (experimental)
An isochronic substitution benefit study of the effects of screen time on the cognitive abilities of 3–6 children (Zhenya et al.)	Teaching and Learning Methods	Quantitative (experimental)
Corrigendum: Ethnocultural empathy development of future language teachers through digital multiliteracy resources for low-literacy adult migrants (Fernández-Corbacho et al. b)	Teacher training	Quantitative (cuasi experimental)
Exploring the factors influencing high school students' deep learning of English in blended learning environments (Shi and Lan)	Teaching and Learning Methods	Quantitative (cuasi experimental)
Prevalence of phubbing behavior in school and university students in Spain (Barbed-Castrejón et al.)	Learning and Psichology	Quantitative (cuasi experimental)
Ethnocultural empathy development of future language teachers through digital multiliteracy resources for low-literacy adult migrants (Fernández-Corbacho et al. a)	Teacher training	Quantitative (cuasi experimental)
Immersive virtual reality for learning about ecosystems: effect of two signaling levels and feedback on action decisions (Porte et al.)	Teaching and Learning Methods	Quantitative (experimental)
Shaping the future of creative education: the transformative power of VR in art and design learning (Serna-Mendiburu and Guerra-Tamez)	Teaching and Learning Methods	Quantitative (cuasi experimental)
Educators as agents of breadth-biased learning: using social reconstructionism as rationale for embracing media multitasking and enhancing teaching practices in higher education (Kassie)	Teacher training	Systematic review
Gamification in the classroom: Kahoot! As a tool for university teaching innovation (Aibar-Almazán et al.)	Teaching and Learning Methods	Quantitative (experimental)
Assessing the intention to accept inquiry-based teaching pedagogy among Chinese university students: an extension of technology acceptance model (Hu et al.)	Teaching and Learning Methods	Quantitative (cuasi experimental)
The effect of self-directed online metacognitive listening practice on Chinese EFL learners' listening ability, metacognition, and self-efficacy (Pei et al.)	Teaching and Learning Methods	Quantitative (experimental)

We have also taken into account a double approach to the research methodology of the respective studies evaluated: quantitative research with a detailed empirical sample and mixed research. In the latter, a qualitative approach complementary to the quantitative approach has also been considered.

Furthermore, as can be seen in Table 1, research based on comprehensive reviews of the state of the research question has also been considered, from which both conclusions and a prospective of the researched topic have been added.

This monograph has made progress on the topics referred to above. This includes the study of digitization and its possibilities of interaction in fields directly or indirectly linked to education. Significant advances in the matter are remarkable. Among these developments we highlight the new possibilities of inclusion enabled by open access tools, the possibilities of gamification as an enhancement of perception and, consequently, the motivation of teaching-learning processes on the basis of common digital tools. Also the necessary digital training of teachers, the new possibilities of mental construction of learning through the use of digitization or the new possibilities of efficiency, transparency and equity of education systems according to their digitization.

As a prospective, these developments have had an updated view from numerous samples of informants in different teaching-learning procedures and their cognitive repercussions. With a view to future research, we consider it highly desirable to investigate areas such as the affective dimension of digitalization and its behavioral repercussions both from the individual and institutional point of view, based on the necessary interaction of educational procedures. We also consider it particularly interesting to investigate the possibilities of access to educational procedures based on digitalization and the hypothetical social consequences of exclusion in the case of lack of such a possibility. This opens the door to traditional alternative procedures and their limitations for social inclusion in the respective modern socio-political systems. The possibilities of exclusion open up the debate about the new educational needs of the population beyond the historically considered needs.

